# Prediction of inpatient mortality in hospitalised children in low- and middle-income countries: An external validation of paediatric mortality risk scores

**DOI:** 10.7189/jogh.14.04235

**Published:** 2024-12-30

**Authors:** Daniella Brals, Ananda Pradhan, Amelie von Saint Andre-von Arnim, Assaf P Oron, Moses Ngari, Narshion Ngao, Ezekiel Mupere, Mohammod J Chisti, Christopher Lwanga, Farzana Afroze, Robert Bandsma, Judd L Walson, James A Berkley, Wieger Voskuijl

**Affiliations:** 1 Amsterdam UMC, location University of Amsterdam, Department of Global Health, Amsterdam Institute for Global Health and Development, Amsterdam, the Netherlands; 2 Departments of Paediatrics and Global Health, University of Washington, Seattle Children’s, Seattle, Washington, USA; 3 Institute for Health Metrics and Evaluation, University of Washington, Seattle, Washington, USA; 4 Childhood Acute Illness and Nutrition Network, Nairobi, Kenya; 5 KEMRI, Wellcome Trust Research Programme, Kilifi, Kenya; 6 Nutrition and Clinical Services Division (NCSD), International Centre for Diarrhoeal Disease Research (icddr,b), Dhaka, Bangladesh; 7 Department of Paediatrics and Child Health School of Medicine College of Health Sciences, Makerere University, Kampala, Uganda; 8 Department of Nutritional Sciences, University of Toronto, Toronto, Ontario, Canada; 9 Translational Medicine Program, The Hospital for Sick Children, Toronto, Ontario, Canada; 10 Departments of Global Health, Medicine, Paediatrics and Epidemiology, University of Washington, Seattle, Washington, USA; 11 Centre for Tropical Medicine and Global Health, University of Oxford, Oxford, UK; 12 Amsterdam UMC, location University of Amsterdam, Amsterdam Institute for Global Child Health, Emma Children’s Hospital, Amsterdam, the Netherlands; 13 Department of Paediatrics and Child Health, Kamuzu University of Health Sciences, Blantyre, Malawi

## Abstract

**Background:**

Risk prediction tools for acutely ill children have been developed in high- and low-income settings, but few are validated or incorporated into clinical guidelines. We aimed to assess the performance of existing paediatric early warning scores for use in low- and middle-income countries using clinical data from a recent large multi-country study in Africa and South-Asia.

**Methods:**

We used data (children across three nutritional strata) from the Childhood Acute Illness and Nutrition (CHAIN) Network cohort study (n = 3101). We assessed 10 scores where similar predictor variables were available in the CHAIN cohort. We evaluated performance using the area under the receiver operating curve (AUC) (primary outcome), sensitivity, specificity, positive and negative predictive value, and positive and negative likelihood ratio (secondary outcomes).

**Results:**

Most scores showed poor discrimination, and all scores had low sensitivity. The paediatric early death index for Africa (AUC = 0.80; 95% confidence interval (CI) = 0.77–0.83), respiratory index of severity in children (AUC = 0.77; 95% CI = 0.74–0.81), and respiratory index of severity in children in Malawi (AUC = 0.78; 95% CI = 0.75–0.82) showed acceptable/good overall discrimination. Among children without wasting, most scores had acceptable/good performance, some even excellent. Poor discrimination was found for most scores among children with moderate and severe wasting or kwashiorkor.

**Conclusions:**

All scores demonstrated lower validation performance than originally reported. Among children without wasting, most risk prediction scores performed acceptably whilst in malnourished children they performed poorly. There is a need for a malnutrition specific score. Further research is needed on specific actions in responding to scores. Integration into future guidelines will require acknowledging staffing, resources and workflows.

Despite considerable reductions in global child deaths over the past decades, child mortality remains high in many low- and middle-income countries (LMICs) [1] with inpatient case fatality from acute illness as high as 10%, largely due to preventable and treatable conditions such as malaria, pneumonia, and diarrheal diseases [2]. Deaths in hospitalised children often occur within the first 24-48 hours of admission [3]. Given the high burden of hospital admissions with health care provider staffing frequently well below World Health Organization (WHO) recommendations [4], a key challenge is how to identify patients at high risk of death in a timely manner to best allocate limited resources and effectively target clinical interventions.

A variety of terminologies exist for signs and syndromes described by WHO and existing prognostic or early warning scores, which can be collectively regarded as clinical warning signs (CWS), such as respiratory distress and reduced consciousness [5]. Clinical warning signs are indicators of disease severity and are used in prognostic or early warning scores for hospitalised children in low-resource settings such as the Lambaréné organ dysfunction score (LODS), Brighton paediatric early warning signs (PEWS), and inpatient triage, assessment, and treatment (ITAT) [6–8]. The rationale behind these scores is that earlier triage could prevent or identify further clinical deterioration and better manage it. Effective triage is particularly important in health care settings with high patient-to-staff ratios, limited infrastructure for the critically ill, and poor or delayed access to higher levels of care, as is the case in many LMICs.

Many published paediatric early warning scores have been limited by methodological concerns [9,10], such as a priori selection of predictors [11], low event rates (i.e. mortality) or bias [8]. Only a few scores developed for mortality risk prediction in LMICs have been robustly validated [12,13] and it has remained challenging to choose the most useful ones. Although early identification and resuscitation of the critically ill can decrease mortality [11,14], none of these scores are routinely used in clinical practice in LMICs [8]. Scores are not typically developed to be integrated with the health care workers’ workflow and often lack actionable recommendations based on the predicted risk [15]. Other barriers to implementation include the complexity of implementing change in multifaceted resource-constrained systems, availability of process improvement components, governance, and administrative components to sustain the change, limitations within health systems (e.g. district or referral hospital and care escalation process), and the perceived origin and complexity of clinical scores [16].

In this study, we aimed to conduct an independent, external validation of existing paediatric prognostic and early warning scores predicting inpatient mortality in LMICs, using data from the Childhood Acute Illness and Nutrition (CHAIN) Network cohort study in Africa and South Asia [17].

## METHODS

### Study design and participants

The CHAIN Network cohort study is described in detail elsewhere [17]. Briefly, participants were children aged two to 23 months with acute illness admitted to nine hospitals in six countries across sub-Saharan Africa and South Asia between 20 November 2016 and 31 January 2019. Participating hospitals were selected to reflect various hospital levels, rural and urban environments and differing prevalences of malaria and HIV. Sites were Dhaka Hospital of icddr,b and Matlab Hospital (Dhaka and Matlab, Bangladesh), Banfora Referral Hospital (Banfora, Burkina Faso), Kilifi County Hospital, Mbagathi County Hospital, and Migori County Referral Hospital (Kilifi, Nairobi, and Migori, Kenya), Queen Elizabeth Central Hospital (Blantyre, Malawi), Civil Hospital (Karachi, Pakistan), and Mulago National Referral Hospital (Kampala, Uganda). 3101 children were enrolled in a 2:1:2 ratio in three strata according to mid-upper arm circumference (MUAC). First stratum referred to no wasting (MUAC≥12.5 cm, age ≥6 months; or MUAC≥12.0 cm, age <6 months), second stratum moderate wasting (MUAC = 11.5–12.5 cm, age ≥6 months; or MUAC = 11.0–12.0 cm, age <6 months), and third stratum severe wasting (MUAC<11.5 cm, age ≥6 months; or MUAC<11.0 cm, age <6 months, or bipedal nutritional oedema (kwashiorkor)). CHAIN deliberately over-recruited children with malnutrition, who are at higher risk of mortality [18–20], and adjusted for this in the analysis by applying population weights.

Ethical approval was obtained from the University of Oxford Tropical Research Ethics Committee and ethics committees in all participating countries. After information was provided in the local language, written informed consent was obtained from all participants’ parents or caregivers.

### Procedures

Upon admission, a full clinical examination and anthropometry were performed by trained study clinicians and pulse oximetry, a full blood cell count, biochemistry, plasma glucose concentration, rapid malaria test [21], and HIV test [22] were systematically conducted. Significant energy and time have been invested a priori in harmonising data collection between sites, with uniform standard operating procedures and case report forms. Harmonisation also included multiple site visits by the paediatrician clinical coordinator.

### Selection of paediatric early warning scores for external validation

We used the following criteria to select scores currently developed for paediatric mortality risk prediction in LMICs: 1) the score was developed for children older than four weeks, 2) used admission predictors only, 3) sufficient information was published to allow calculation of individual risk scores, and 4) identical or very similar predictor variables were available in the CHAIN cohort to enable accurate mapping of the score. [REMOVED HYPERLINK FIELD]Based on these criteria, we selected 10 paediatric early warning scores: LODS [8], family-assisted severe febrile illness therapy (FASTER) [23], temperature, oxygen saturation, pulse rate, respiratory rate, sensorium and seizures (TOPRS) [24], ITAT [6], WHO emergency signs [5], Mpimbaza et al. [25], PEWS [26], paediatric early death index for Africa (PEDIA) (early and late) [27], respiratory index of severity in children (RISC) (only HIV-negative) [28], and RISC-Malawi [29] (Table S1 in the **Online Supplementary Document**). Scores that we considered but did not select as several predictor variables were not available in the CHAIN cohort, included fluid as expansive supportive therapy-paediatric emergency triage, fluid as expansive supportive therapy-paediatric emergency triage with additional laboratories [12], signs of inflammation that can kill [30,31], PEWS for resource-limited settings [32], paediatric advance warning score [33], pneumonia aetiology research for child health (PERCH) [34], Lowlavaar et al. [35], Erdman et al. [36], and Paediatric risk of mortality III [37].

For some scores, we needed to make adaptations to the original risk prediction score due to variable differences from the CHAIN cohort (Table S1 in the **Online Supplementary Document**). In CHAIN, weak pulse and low or unmeasurable blood pressure were not collected; hence, these were not included to construct the variable ‘shock’ in the WHO emergency signs score. Age-specific heart rates were used to construct the variable shock in the WHO emergency signs score [38] because no heart rate ranges were provided in the original paper [5]. In order to calculate the Brighton PEWS score, we did not include all circulation and respiratory variables due to data limitations in CHAIN (Table S1 in the **Online Supplementary Document**). Capillary refill time in the CHAIN data distinguished between <2 seconds, 2–3 seconds, and >3 seconds, whereas the Brighton PEWS distinguished three seconds (plus one point), four seconds (plus two points), and ≥5 seconds (plus three points). Therefore, we added one point if the capillary refill time was 2–3 seconds and two points if this was >3 seconds. Finally, as admission for surgery was an exclusion criterion in CHAIN, post-operative vomiting always scored zero points.

### Outcomes

For each selected score, we evaluated performance in predicting inpatient mortality, using the area under the receiver operating curve (AUC) (primary outcome) and sensitivity, specificity, positive and negative predictive value, positive and negative likelihood ratio results (secondary outcomes). To be able to compare the prediction performance between scores we focused on predicting inpatient mortality only, including when the risk prediction score was designed to predict clinical deterioration instead of mortality. In sensitivity analysis we also evaluate mortality at set time points (two days, five days, seven days, and 30 days).

### Statistical analysis

We performed data cleaning and analyses using Python, version 3.9.7 (Python Software Foundation, Wilmington, Delaware, USA), with the ‘pandas,’ ‘numpy,’ ‘sklearn metrics,’ ‘matplotlib pyplot,’ and ‘plotnine’ libraries. AUCs were classified according to Mandrekar (i.e. AUC = 0.8–1.0 for excellent discrimination, AUC = 0.7–0.8 for acceptable/good discrimination, AUC = 0.5–0.7 for poor discrimination, and AUC<0.5 as not useful) [39].

We adjusted the analysis for the intentional over-representation of malnourished children in the CHAIN cohort by applying population weights [40]. First the weighted AUC, including 95% confidence intervals (CIs), sensitivity, specificity, positive predictive value, negative predictive value, positive and negative likelihood ratios, were calculated using the full CHAIN cohort. Subsequently, unweighted AUCs and CIs were calculated within each of the three CHAIN strata – no wasting, moderate wasting, and severe wasting or kwashiorkor.

Three mortality risk scores (PEDIA early and late, RISC, and RISC-Malawi) did not have a predefined cut-off for increased mortality risk, in which case the statistically optimal cut-off was determined by using Youden’s J statistic [41]. We first tested scores designed explicitly for a population with a specific disease (e.g. children with malaria) in the entire CHAIN cohort and subsequently in the population for which the score was designed. In sensitivity analyses, we investigated site and age variations by repeating the analysis after stratifying cases according to the nine study sites and age groups (two to six months, six to 12 months, 12–18 months, and 18–24 months), respectively.

## RESULTS

Between 20 November 2016 and 31 January 2019, 3101 children were enrolled in the CHAIN cohort, of whom 1120 (36.1%) had no wasting, 763 (24.6%) had moderate wasting, and 1218 (39.3%) had severe wasting or kwashiorkor [42]. 1721 (55%) children were admitted for diarrhoea, 667 (21%) for severe pneumonia, 440 (14%) for malaria, and 443 (14%) for severe anaemia. Mortality in the CHAIN cohort was 3.5% for the children with no wasting, 8.1% in the moderate wasting group and 20.4% for children with severe wasting or kwashiorkor [17]. Participant characteristics are shown by the three nutritional strata in the original publication [17].

**Figure 1:**
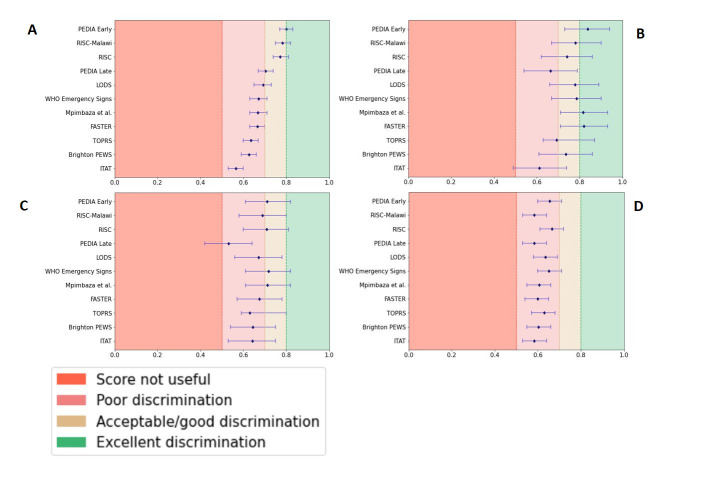
AUCs with 95% confidence intervals for the 10 validated paediatric early warning scores for all children (full CHAIN cohort; weighted AUCs) and within the three nutritional strata (unweighted AUCs). **Panel A.** All children. **Panel B.** No wasting. **Panel C.** Moderate wasting. **Panel D.** Severe wasting or kwashiorkor. AUC – area under the receiver operating curve.

Most scores showed poor discrimination when applied to the weighted CHAIN cohort (**Figure 1**, Panel A, **Table 1**), only the RISC, RISC-Malawi, and PEDIA early showed acceptable/good discrimination, with weighted AUCs of 0.78 (95% CI = 0.74–0.81), 0.77 (95% CI = 0.73–0.8), and 0.72 (95% CI = 0.68–0.76), respectively. These three scores plus PEDIA late all contain malnutrition parameters (weight for age Z-score (WAZ) and MUAC), while others did not. None of the scores attained excellent performance. Most scores had a high specificity and high negative predictive value but a low sensitivity and low positive predictive value in the weighted CHAIN cohort (**Table 1**). Weighted sensitivity ranged from 73% to 19%, with a median of 53%. Weighted positive-predictive values ranged from 28% to 4%, with a median of 8%.

**Table 1:** Weighted results for the 10 validated paediatric early warning scores*

Risk prediction tool	Range score	Cut-off for increased mortality risk	AUC (95% CI)	Sensitivity (%)	Specificity (%)	True positives (n)	False positives (n)	True negatives (n)	False negatives (n)	PPV (%)	NPV (%)	LR (+)	LR (–)	Deaths (n)†
PEDIA early	0–9	3	0.80 (0.77–0.83)	0.93	0.50	218	2909	2852	16	6.97	99.44	1.84	0.14	234
RISC-Malawi	0–23	7	0.78 (0.75–0.82)	0.68	0.79	159	1182	4579	75	11.86	98.39	3.31	0.40	234
RISC	0–6	3	0.77 (0.74–0.81)	0.58	0.85	135	890	4871	99	13.17	98.01	3.73	0.50	234
PEDIA late	0–7	3	0.70 (0.67–0.74)	0.74	0.63	173	2114	3647	61	7.56	98.35	2.01	0.41	234
LODS	0–3	1	0.69 (0.65–0.73)	0.50	0.86	116	801	4960	118	12.65	97.68	3.57	0.59	234
WHO emergency signs	0–7	1	0.67 (0.63–0.71)	0.57	0.70	134	1754	4007	100	7.10	97.57	1.88	0.61	234
Mpimbaza et al.	0–14	5	0.67 (0.63–0.71)	0.26	0.97	60	157	5604	174	27.65	96.99	9.41	0.76	234
FASTER	0–3	2	0.67 (0.63–0.70)	0.53	0.76	124	1378	4383	110	8.26	97.55	25.85	0.83	234
TOPRS	0–6	2	0.64 (0.60–0.67)	0.73	0.47	170	3039	2722	64	5.30	97.70	1.82	0.76	234
Brighton PEWS	0–13	4	0.63 (0.59–0.66)	0.63	0.45	147	3186	2575	87	4.41	96.73	1.14	0.83	234
ITAT	0–8	4	0.57 (0.53–0.6)	0.19	0.85	44	879	4882	190	4.77	96.25	1.23	0.96	234

AUC – area under the receiver operating curve, CI – confidence interval, FASTER – family-assisted severe febrile illness therapy, ITAT – inpatient triage, assessment, and treatment, LODS – Lambaréné Organ dysfunction score, LR (+) – positive likelihood ratio, LR (–) – negative likelihood ratio, NPV – negative predictive value, PEDIA – paediatric early death index Africa, PEWS – paediatric early warning scores, PPV – positive predictive value, RISC – respiratory index of severity in children, TOPRS – temperature, oxygen saturation, pulse rate, respiratory rate, sensorium, and seizures, WHO – World Health Organization

*Data are ordered from largest to smallest based on the weighted AUCs.

†Sample n = 5995.

**Figure 2:**
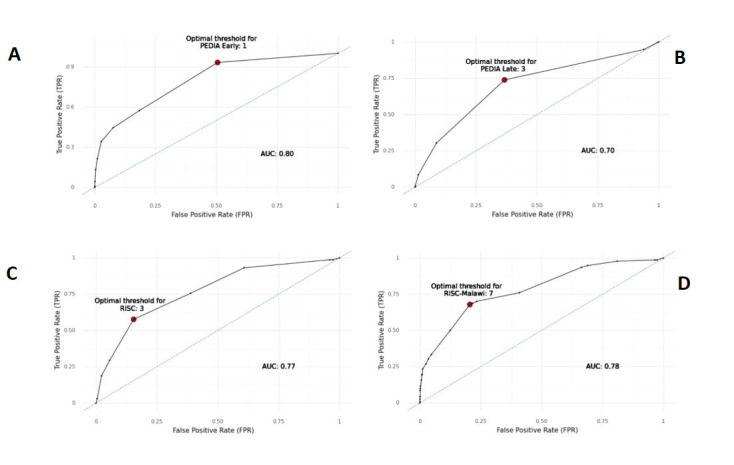
Optimal cut-off based on Youden’s J statistic [39]. **Panel A.** PEDIA early. **Panel B.** PEDIA late. **Panel C.** RISC. **Panel D.** RISC-Malawi.

Among the children without wasting, most scores had acceptable/good performance, some excellent (PEDIA early AUC = 0.84, 95% CI = 0.73–0.94; Mpimbaza et al. AUC = 0.82, 95% CI = 0.71–0.93; and FASTER AUC = 0.82, 95% CI = 0.71–0.93) and only three scores showed poor discrimination (PEDIA late, TOPRS, and ITAT) (**Figure 1**, Panels B–D). On the other hand, we found poor discrimination for almost all scores when applied to children with moderate and severe wasting or kwashiorkor, where the scores performed worst among children with severe wasting or kwashiorkor. For example, the PEDIA early score, the best-performing score in the full CHAIN cohort, and a score that includes WAZ as a predictor showed poor discrimination when applied only to children with severe wasting or kwashiorkor, while its performance was still acceptable/good among the moderate wasting and no wasting strata. In the moderate wasting stratum, PEDIA early and RISC-Malawi still performed as acceptable/good but with lower AUCs than among the full CHAIN cohort. The WHO emergency signs also performed acceptable/good among the moderate wasting, though only barely, whereas this score showed poor performance in the full CHAIN cohort and the severe wasting or kwashiorkor strata. **Figure 2** shows the cut-offs for the scores without a predefined cut-off for increased mortality risk. We determined the following statistically optimal cut-offs: 1) one for PEDIA early, 2) three for PEDIA late and RISC, and 3) seven for RISC-Malawi.

We calculated disease-specific performance metrics for scores designed to address those diseases. When we restricted the analysis to children with malaria only, the LODS achieved acceptable/good performance (AUC = 0.77; 95% CI = 0.69–0.85), as opposed to poor discrimination in the full CHAIN cohort. When we restricted the RISC and RISC-Malawi scores to children with lower respiratory tract infection only, the scores performed acceptable/good performance (AUC = 0.75; 95% CI = 0.69–0.81) and excellent (AUC = 0.80; 95% CI = 0.75–0.85), respectively.

Figures S1–2 in the **Online Supplementary Document** show the results of the sensitivity analysis, which shows that the performance of the paediatric early warning scores substantially vary by site (country) but not by age group. When analysing performance of the scores in predicting mortality at fixed time points (day two, five, seven, and 30) instead of in-patient mortality only, the top three best performing scores remained similar (PEDIA early, RISC-Malawi, and RISC). AUCs did decrease, though, with performance at day two being better than for mortality prediction at day 30 (Figure S3 in the **Online Supplementary Document**).

## DISCUSSION

We undertook external validation of paediatric early prognostic scores using data from an African and South-Asian data set reflecting a range of hospital levels, urban and rural environments, and different disease prevalences. We tried to include as many paediatric risk prediction scores as possible, and from 21 scores identified in a recent systematic review [10] and from additional literature search, we were able to validate 10 externally. Data from the CHAIN cohort study was rich, well-sourced, harmonised, and used a carefully planned and monitored data collection system [17]. However, half of the scores were not transferable to the data collected in CHAIN. This indicates the challenge of implementing scores across a variety of LMIC settings. As expected, all 10 evaluated scores performed worse than in their original publications. Overall, only the PEDIA early, RISC (among the HIV-negative), and RISC-Malawi showed acceptable/good discrimination. However, among children without wasting, most risk prediction scores were acceptable/good or excellent (PEDIA early, Mpimbaza et al., and FASTER). On the other hand, among children with moderate or severe wasting or kwashiorkor, mortality was higher [17], but scores performed more poorly.

Overall, the best performing three scores in assessing mortality prediction irrespective of nutritional status were PEDIA early, RISC-Malawi, and RISC which all incorporate malnutrition indicators (like WAZ or MUAC). MUAC is known to be associated with mortality [17] although it does not identify pathophysiologic mechanisms. However, when applied to children with severe malnutrition or kwashiorkor only, all three scores performed poorly (**Figure 1**). This suggests that the optimal set of predictors for mortality prediction in children with severe malnutrition or kwashiorkor has not yet been identified. A few predictors for mortality in children with severe acute malnutrition have been identified from Ethiopia (oxygen below 90% saturation, impaired consciousness at admission, intake of formula 100, HIV/AIDS, oedema, and failed appetite test) and Zambia and Zimbabwe (oedema, low MUAC, baseline infections, shock, and lack of home sanitation) [43]. Severe malnutrition may mean that clinical signs are less apparent [44]. Or it may also mean that for signs that are present, mortality is higher when accompanied by severe malnutrition.

Alternatives to scores calculated at admission include the Brighton PEWS [7] and a daily score specifically developed for children with severe malnutrition [45]. However, these have a relatively short period of effective prediction (approximately 48 hours) and still need external validation in LMICs. In the high-income settings, implementation of the bedside PEWS compared with the standard of care did not significantly decrease all-cause mortality among hospitalised paediatric patients [46]. Reasons for this might be the relatively low mortality in the high-income setting or the relatively little impact of additional care. In LMICs settings, limited staffing and lack of dedicated paediatric intensive care unit facilities combined with the absence of actionable recommendations based on the predicted risk [15] might also limit the effectiveness of the efferent response to the score. Thus, how to integrate paediatric early warning scores in clinical workflows and whether they can alter clinical outcomes remain important outstanding questions.

The scores’ performance differed across the different CHAIN sites (Figure S1 in the **Online Supplementary Document**). In Kilifi (Kenya), with a long history of clinical research, including developing prognostic scores, many scores had an AUC of 0.95 while other sites had lower scores. This site-specificity may reflect differences in case mix, resources available and the level of training of score users. This is in line with a recent paper from Agulnik et al. on barriers to implementation of early warning scores that include the complexity of implementing change in a multifaceted health care system [16]. Factors involved mentioned in this paper are inadequate resources affecting the afferent and efferent components of the score, the availability of process improvement components, governance, and administrative components to sustain the change, limitations within health systems (e.g. district or referral hospital and care escalation process), and the perceived origin and complexity of clinical scores [16]. To overcome these barriers, clinicians dealing with sick children would be most aided by a tool which has a limited number of easily measured clinical signs, is highly discriminative for poor outcomes such as mortality, and is responsive to changes in clinical status following therapeutic interventions [11]. Recommendations for a successful implementation of a risk prediction tool in clinical practice should contain an ‘actionable recommendation’ to the predicted risk and the presentation of the risk should be smoothly integrated with the clinician’s workflow [15]. Moreover, attempts to provide health care workers in LMICs with a simple bedside score are not new [24,47] but these scoring systems were constructed for use on initial admission only. This means there is a need for risk prediction scores that are applicable for the duration of hospital admission, including at discharge. We foresee that a bundle-implementation of a score, together with an (emergency) response arm, combined with recurrent health care provider education regarding appropriate use of the score and governance and establishment of a safety culture could be a first step and important step in paediatric departments in LMICs.

There were several limitations in this study. We were able to only externally validate 10 scores using the CHAIN cohort, and not all the 21 existing scores, as predictors such as blood pressure, were not collected in the CHAIN cohort. Even among some of the evaluated scores, minor modifications to the original scoring system had to be made, given the available data in the CHAIN cohort data set. There also were some difficulties in evaluating several of the scores. In addition to original methodological issues, some of the scores could be interpreted differently. Three scores (PEDIA, RISC, and RISC-Malawi) did not have single cut-offs for increased mortality risk, so we calculated those using Youden’s J statistic [41], which may not be clinically optimal. Some scores required laboratory measurements which pose difficulties to implementation as well as external validation [10]. An epidemiological issue common to all these scores is the lack of assessment over a fixed time period. Inpatient mortality may seem instinctively valuable to clinicians. However, variable discharge practices between different studies, hospitals, clinicians (including when to discharge) and diseases, and the fact that a significant number of deaths are observed to occur shortly after discharge [17] likely introduce bias. For similar reasons that a standardised period of assessment is preferable to describe hospital performance, we believe evaluation over a relevant fixed period should be considered a gold standard for assessing prognostic and early warning scores, as was done for the evaluation of PEWS for example (i.e. transfer to intensive care unit) [48,49]. For this reason, we also provide performance assessments for the scores we validated over two, five, seven, and 30 days to allow future comparisons. This did not change the top three best-performing early warning scores. Strengths of the current study are its thorough methodology and the use of a large multi-country data set from LMICs, reflecting urban and rural environments and different disease prevalences and nutritional strata [17].

## CONCLUSIONS

Based on the performance of the scores in this external validation, we propose that the best overall performing score is PEDIA early. Nevertheless, the performance of PEDIA early in wasted children was poor, like all other scores assessed. The findings from a recent qualitative study on the implementation of PEWS in LMICs paediatric oncology hospitals can be used to guide clinicians on strategies to implement evidence-based interventions like risk prediction scores in resource-limited hospitals to improve patient outcomes [16].

Further research approaches should focus on specific action to be taken as a result of timing (at what time during admission do clinical warning signs appear/disappear) of scoring (starting oxygen, transfer to a paediatric intensive care unit, broad-spectrum antibiotics, etc.), and how this may be aligned with resources available and workflow patterns. Machine learning [50] and (simple) biomarkers might aid mortality prediction or guidance for specific therapies such as antimicrobials. Ideally, this would be done near-bedside (point of care test). Another perspective would be to focus on children at very low risk of mortality, as recently done in children with pneumonia [13], to free up bed space, staffing and resources to better care for higher-risk children. Finally, as risk prediction scores performed poorly in severely malnourished children, future risk prediction tools need to take severe malnutrition into account, or even better, a separate score for severely malnourished children will be developed.

## Additional material


Online Supplementary Document
