# Supplementary material for: Prediction of inpatient mortality in hospitalised children in low- and middle-income countries: An external validation of paediatric mortality risk scores
Source: J Glob Health. 2024 Dec 30;14:04235. doi: 10.7189/jogh.14.04235 (PMC11683851; doi:10.7189/jogh.14.04235)
Supplement: Online Supplementary Document [file jogh-14-04235-s001.pdf]

**Table S1. Overview pediatric risk prediction tools and adaptations made in the external validation.**

*Data shown for ≤2 yrs. only, as CHAIN did not include children beyond 2 yrs. HIC = high-income countries. MIC = middle-income countries. Blue text gives alternatives used in CHAIN. Red striped through text shows variables not available in CHAIN.

| Pediatric risk prediction tool | Study population | Outcome | Included clinical signs | Value if present | Similar variables in CHAIN |
|---|---|---|---|---|---|
| Lambaréné Organ Dysfunction Score (**LODS**) (8) | African children with *P. falciparum malaria* | Inpatient mortality | Coma: | | |
| | | | Blantyre coma score ≤2 (2) | 1 | P/U on AVPU-scale (5) |
| | | | Prostration: | | |
| | | | Not being able to breast-feed/sit/stand/walk, depending on age child | 1 | Hypotonic/Lethargic |
| | | | Deep breathing | 1 | Acidotic breathing |
| Max. score | | | | 3 | |
| Cut-off | | | | >0 | |
| Family-Assisted Severe Febrile Illness Therapy (**FASTER**) (21) | Kenyan children with febrile illness | Deterioration within first 24 hrs. | Chest retractions | 1 | |
| | | | Capillary refill >3 sec | 1 | |
| | | | Impaired consciousness: | | |
| | | | P/U on AVPU-scale (5) | 1 | |
| Max. individual score | | | | 3 | |
| Cut-off for increased mortality risk | | | | 1 = yellow flag, 2+ = red flag | |
| Temperature, Oxygen saturation, Pulse rate, Respiratory rate, Sensorium and seizures (**TOPRS**) (22) | Indian children | Emergency room admission mortality | Oxygen saturation <90% | 1 | |
| | | | Temperature: <36 OR >38°C | 1 | |
| | | | Heart rate: | | |

| | | | | | |
|---|---|---|---|---|---|
| | | | Infant | | |
| | | | >160/min | 1 | |
| | | | Child | | |
| | | | >150/min | 1 | |
| | | | Respiratory rate: | | |
| | | | Infant | | |
| | | | >60/min | 1 | |
| | | | Child | | |
| | | | >50/min | 1 | |
| | | | Loss of sensorium | 1 | P/U on AVPU-scale (5) |
| | | | Seizures | 1 | |
| Max. individual score | | | | 6 | |
| Cut-off for increased mortality risk | | | | >1 | |
| Inpatient Triage, Assessment and Treatment (**ITAT**) (22) | Malawian children <15 years | Inpatient mortality | Oxygen saturation: | | |
| | | | <85% | 2 | |
| | | | 85-95% | 1 | |
| | | | Temperature: | | |
| | | | <35 OR >39°C | 2 | |
| | | | 35-35.9 OR 37.5-39°C | 1 | |
| | | | Heart rate: | | |
| | | | <3 months | | |
| | | | <90 OR >180/min | 2 | |
| | | | 90-109 OR 151-180/min | 1 | |
| | | | 3-12 months | | |
| | | | <80 OR >170/min | 2 | |
| | | | 80-99 OR 151-170/min | 1 | |
| | | | 12-24 months* | | |
| | | | <70 OR >150/min | 2 | |
| | | | 70-89 OR 121-150/min | 1 | |

| | | | | | |
|---|---|---|---|---|---|
| | | | Respiratory rate: | | |
| | | | <ins><3 months</ins> | | |
| | | | <20 OR > 80/min | 2 | |
| | | | 20-29 OR 61-80/min | 1 | |
| | | | <ins>3-12 months</ins> | | |
| | | | <20 OR >70/min | 2 | |
| | | | 20-24 OR 51-70/min | 1 | |
| | | | <ins>12-24 months</ins>* | | |
| | | | <15 OR >60/min | 2 | |
| | | | 15-19 OR 41-60/min | 1 | |
| Max. individual score | | | | 8 | |
| Cut-off for increased mortality risk | | | | >3 | |
| WHO Emergency Signs (5) | Children in general | Clinical deterioration | Obstructed/Absent breathing | 1 | Wheezing |
| | | | Severe respiratory distress | 1 | Any 2 of the following: Grunting/Chest indrawing/Nasal flaring/Acidotic breathing |
| | | | Central cyanosis | 1 | |
| | | | Signs of shock | 1 | |
| | | | Cold hands + capillary refill >3 sec + high heart rate + weak pulse + low/unmeasurable blood pressure | | Cold hands/Shoulders/Elbows + capillary refill >3 sec + high age specific heart rate ~~+ weak pulse + low/unmeasurable blood pressure~~ |
| | | | Impaired consciousness | 1 | |
| | | | P/U on AVPU-scale (5) | | |
| | | | Convulsions | 1 | |
| | | | Severe dehydration + diarrhea | 1 | |
| | | | Severe dehydration = lethargy/sunken eyes/very slow return after pinching the skin | | |
| Max. individual score | | | | 7 | |
| Cut-off for increased mortality risk | | | | >0 | |
| Brighton Pediatric Early | HIC/MIC children <16 years old | Clinical deterioration | *Behavior/disability* | | |

| | | | | | |
|---|---|---|---|---|---|
| Warning Score (**PEWS**) (24) | | | | | |
| | | | U on AVPU-scale (5) | 3 | |
| | | | P on AVPU-scale (5) | 2 | |
| | | | V on AVPU-scale (5) | 1 | |
| | | | *Circulation* | | |
| | | | Grey/Central cyanosis + mottled OR Capillary refill ≥5 sec OR Tachycardia of 30 beats/min above normal rate OR Bradycardia | 3 | ~~Grey/~~Central cyanosis ~~+ mottled~~ OR ~~Capillary refill ≥5 sec~~ OR Tachycardia of 30 beats/min above normal rate OR Bradycardia |
| | | | Grey/Central cyanosis OR Capillary refill 4 sec OR Tachycardia of 20-30 beats/min above normal rate | 2 | ~~Gery/~~Central cyanosis OR Capillary refill >3 sec OR Tachycardia of 20-30 beats/min above normal rate |
| | | | Pale OR Capillary refill 3 sec | 1 | ~~Pale OR~~ Capillary refill 2-3 sec |
| | | | *Respiratory* | | |
| | | | Respiratory rate ≥30 above normal parameters OR Respiratory rate ≤5 below normal parameters OR Retractions/Grunting OR $Fi0_2$ >0.3 (CPAP/BiPAP) OR ≥2 L/min $O_2$ | 3 | Respiratory rate ≥30 above normal parameters OR Respiratory rate ≤5 below normal parameters OR Retractions/Grunting ~~OR $Fi0_2$ >0.3 (CPAP/BiPAP) OR ≥2 L/min $O_2$~~ |
| | | | Respiratory rate ≥20 above normal parameters OR Jugular retractions OR $Fi0_2$ >0.4 (CPAP/BiPAP) OR ≥5 L/min $O_2$ | 2 | Respiratory rate ≥20 above normal parameters ~~OR Jugular retractions OR $Fi0_2$ >0.4 (CPAP/BiPAP) OR ≥5 L/min $O_2$~~ |
| | | | Respiratory rate ≥10 above normal parameters OR Retractions OR $Fi0_2$ >0.3 (CPAP/BiPAP) OR ≥2 L/min $O_2$ | 1 | Respiratory rate ≥10 above normal parameters ~~OR Retractions OR Fi02 >0.3 (CPAP/BiPAP) OR ≥2 L/min~~ |
| | | | Continuous administration of inhalation medications/CPAP treatments | 2 | ~~Continuous administration of inhalation medications or continuous positive airway pressure (CPAP) treatments~~ |
| | | | Presence of persistent postoperative vomiting | 2 | ~~Presence of persistent postoperative vomiting~~ |
| Max. individual score | | | | 13 | |
| Cut-off for increased mortality risk | | | | >3 OR >2 within one of the sub domains (behavior, circulation, respiratory) | |
| Mpimbaza et al. (23) | Ugandan children | Inpatient mortality | *Signs* | | |
| | | | Age ≤4 months | 2 | |

| | | | | | |
|---|---|---|---|---|---|
| | | | No subjective fever | 1 | |
| | | | Difficulty breathing | 1 | |
| | | | Altered consciousness | 1 | |
| | | | Unable to drink/breastfeed | 1 | |
| | | | Convulsions | 1 | |
| | | | *Symptoms* | | |
| | | | Temperature ≤ 35.5°C | 1 | |
| | | | Pallor | 1 | Anemia |
| | | | Jaundice | 1 | |
| | | | Deep breathing | 1 | Acidotic breathing |
| | | | Unconscious | 1 | P/U on AVPU-scale (5) |
| | | | Unable to sit up or stand | 1 | Hypotonic |
| | | | Signs of meningitis | 1 | Lethargic/Irritable/Bulging fontanelle |
| Max. individual score | | | | 14 | |
| Cut-off for increased mortality risk | | | | >4 | |
| **PEDIA Early** (25) | Kenyan children >90 days old | Inpatient mortality within 48 hrs. | Jaundice | 1 | |
| | | | Lower chest wall indrawing | 1 | |
| | | | Prostrated + Seizures | 2 | Hypotonic/Lethargic + Seizures |
| | | | Prostrated + No seizures | 2 | Hypotonic/Lethargic + No seizures |
| | | | Impaired consciousness + Seizures | 2 | P/U on APVU-scale (5) + Seizures |
| | | | Impaired consciousness + No seizures | 3 | P/U on APVU-scale (5) + No seizures |
| | | | Weight for age z-score <-3 | 1 | |
| | | | Kwashiorkor | 1 | Nutritional oedema |
| Max. individual score | | | | 9 | |
| Cut-off for increased mortality risk | | | | Not provided | |
| **PEDIA Late** (25) | Kenyan children >90 days old | Inpatient mortality after 48 hrs. | Constant | 2 | |
| | | | History of illness of more than 7 days | 1 | Admitted in past month/Presenting with >14 days cough/Presenting with >14 days |

| | | | | | |
|---|---|---|---|---|---|
| | | | Prostrated + Seizures | -1 | Hypotonic/Lethargic + Seizures |
| | | | Prostrated + No seizures | 0 | Hypotonic/Lethargic + No seizures |
| | | | Impaired consciousness + Seizures | 1 | P/U on APVU-scale (5) + Seizures |
| | | | Impaired consciousness + No seizures | 1 | P/U on APVU-scale (5) + No seizures |
| | | | Temperature (°C) | | |
| | | | <36 | 1 | |
| | | | >39 | -1 | |
| | | | Weight for age z-score <-3 | 1 | |
| | | | Kwashiorkor | 1 | Nutritional oedema |
| Max. individual score | | | | 7 | |
| Cut-off for increased mortality risk | | | | Not provided | |
| Respiratory Index of Severity in Children (**RISC**) **HIV-negative** (26) | South African children with lower respiratory tract infection | Inpatient mortality | IF Oxygen saturation (%) | | |
| | | | <90 | 3 | |
| | | | ELSE IF Chest indrawing | 2 | |
| | | | Wheezing | -2 | |
| | | | Refusing feedings | 1 | |
| | | | Weight for age z-score | | |
| | | | <-3 | 2 | |
| | | | >-3 and ≤-2 | 1 | |
| Max. individual score | | | | 6 | |
| Cut-off for increased mortality risk | | | | Not provided | |
| Malawi RSIC (**mRISC**) (27) | Malawian children | Inpatient mortality | Oxygen saturation (%) | | |
| | | | <90 | 7 | |
| | | | 90-92 | 2 | |
| | | | MUAC (cm) | | |
| | | | <11.5 | 7 | |
| | | | 11.5-13.5 | 3 | |
| | | | Female | 1 | |

| | | | |
|---|---|---|---|
| | Wheezing | -2 | |
| | Unconscious | 8 | P/U on AVPU-scale (5) |
| Max. individual score | | 23 | |
| Cut-off for increased mortality risk | | Not provided | |

*Data shown for ≤2 yrs. only, as CHAIN did not include children beyond 2 yrs. HIC = high-income countries. MIC = middle-income countries. Blue text gives alternatives used in CHAIN. Red striped through text shows variables not available in CHAIN.

**Figure S1. Weighted AUCs with 95%CI for the 10 validated pediatric early warning scores, by study site**

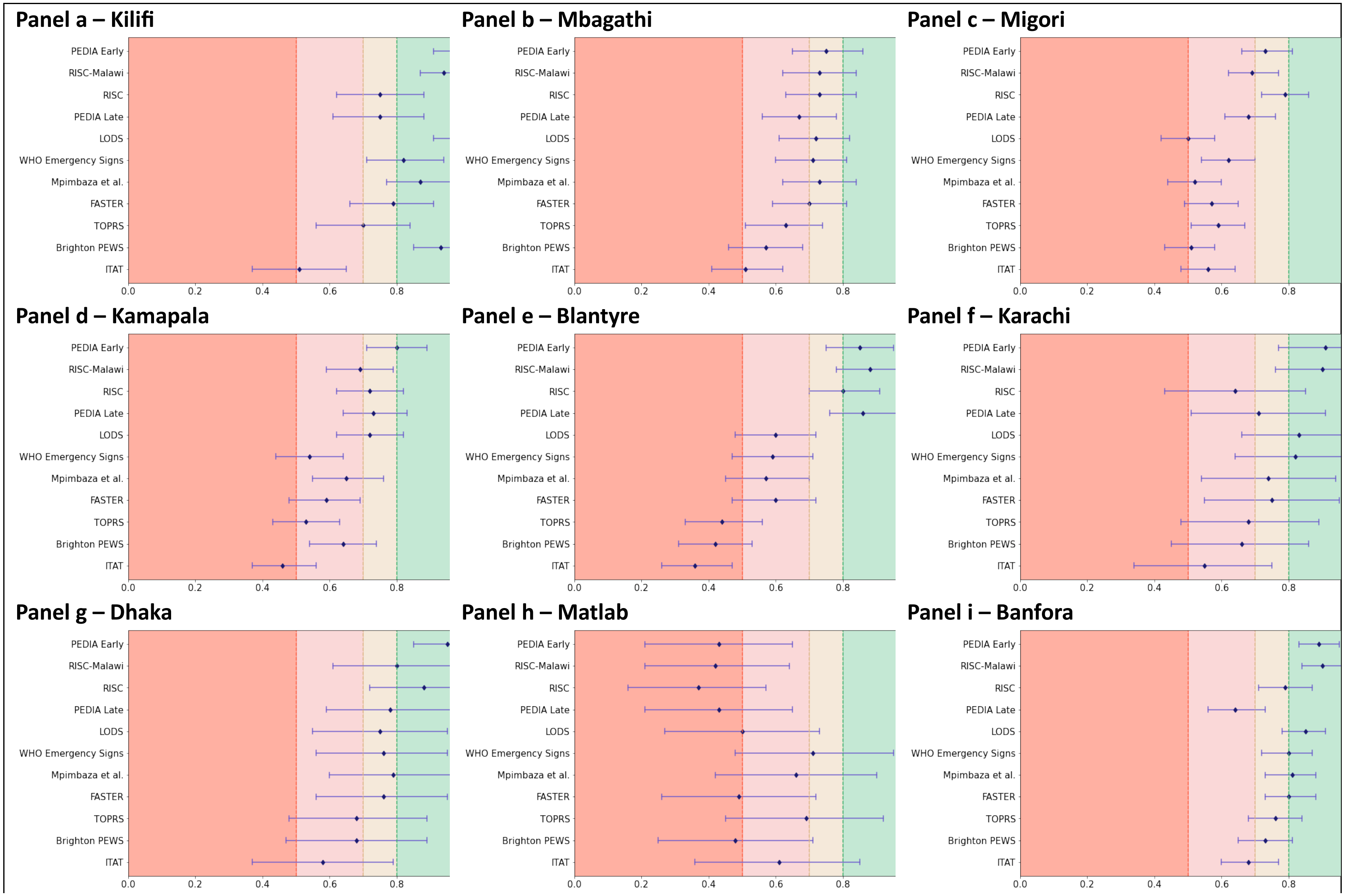

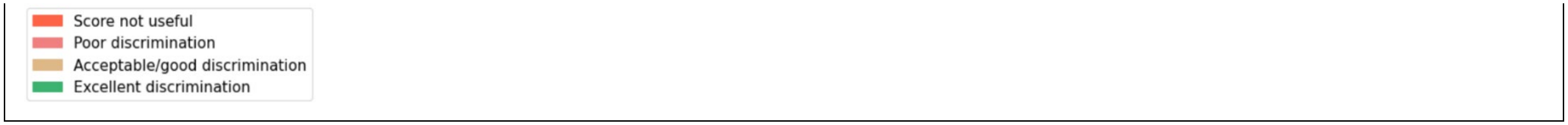


AUC = area under the receiver operating curve. AUCs are denoted by the diamond, 95%CIs are shown by the bars around the AUC.

**Figure S2. Weighted AUCs with 95%CI for the 10 validated pediatric early warning scores, by age group**

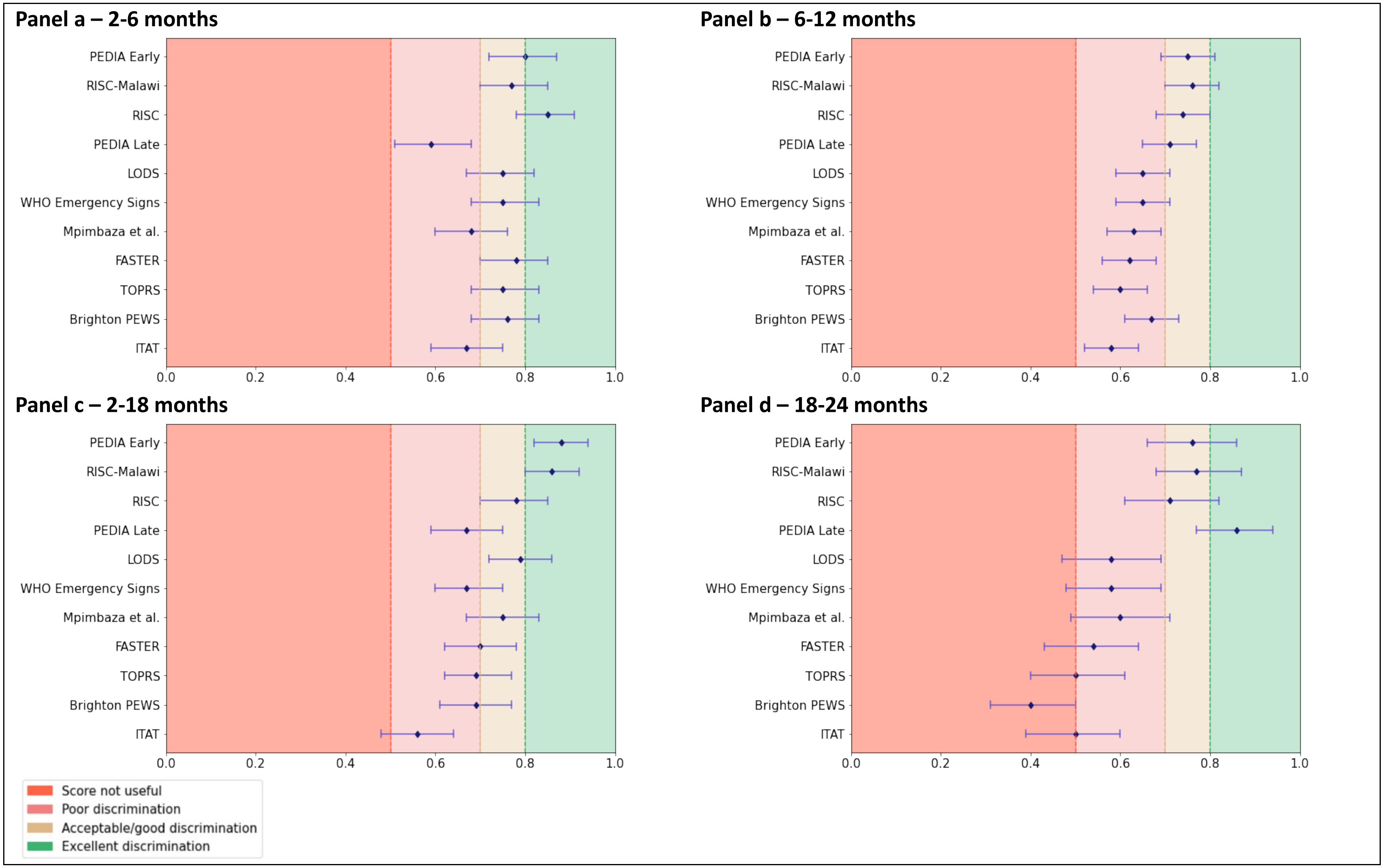


AUC = area under the receiver operating curve. AUCs are denoted by the diamond, 95%CIs are shown by the bars around the AUC.

**Figure S3. Weighted AUCs with 95%CI for the 10 validated pediatric early warning scores to predict 2-, 3-, 7-, and 30-day mortality (instead of inpatient mortality)**

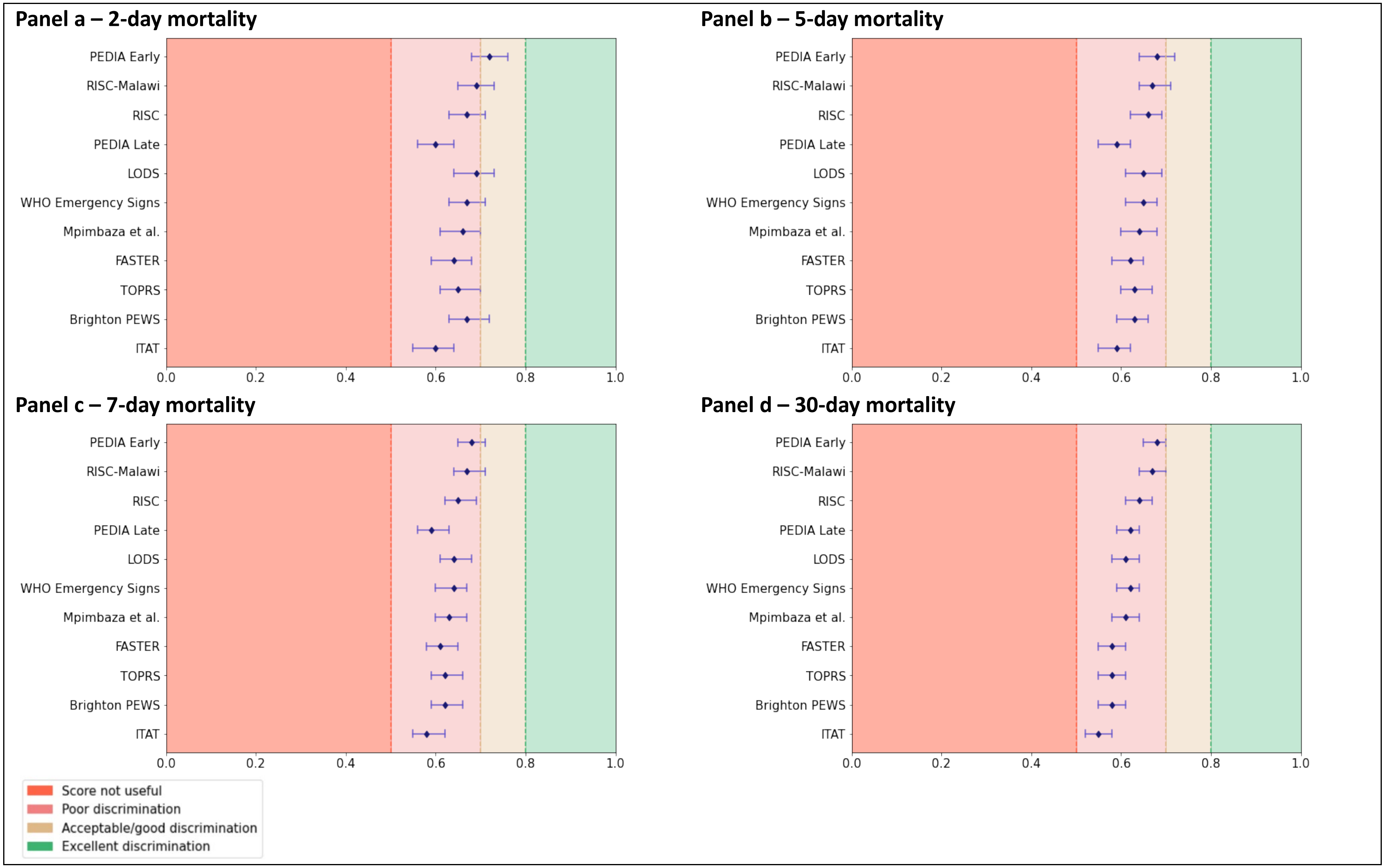


AUC = area under the receiver operating curve. AUCs are denoted by the diamond, 95%CIs are shown by the bars around the AUC.
